# A Comprehensive Analysis of Novel Variations Associated with Bile Duct Cancer: Insights into Expression, Methylation, and 3D Protein Structure

**DOI:** 10.3390/ijms262311244

**Published:** 2025-11-21

**Authors:** Alper Bülbül, Gizel Gerdan, Cansu Portakal, Sudenaz Bajrami, Cemaliye Boylu Akyerli

**Affiliations:** 1 Department of Biostatistics and Bioinformatics, Institute of Health Sciences, Acıbadem Mehmet Ali Aydınlar University, İstanbul 34638, Türkiye; 2 Department of Genome Studies, Institute of Health Sciences, Acıbadem Mehmet Ali Aydınlar University, İstanbul 34638, Türkiye; gizel.gerdan@live.acibadem.edu.tr (G.G.); cansu.portakal@live.acibadem.edu.tr (C.P.); 3 Department of Molecular Biology and Genetics, Faculty of Engineering and Natural Sciences, Acıbadem Mehmet Ali Aydınlar University, İstanbul 34638, Türkiye; sudenaz.bajrami@live.acibadem.edu.tr; 4 Department of Medical Biology, School of Medicine, Acıbadem Mehmet Ali Aydınlar University, İstanbul 34638, Türkiye

**Keywords:** bile duct cancer, cholangiocarcinoma, somatic variation analysis, multi-omics

## Abstract

Cholangiocarcinoma is a rare but highly lethal cancer of the biliary epithelium, marked by heterogeneous molecular subtypes, unclear etiology, and poor five-year survival, highlighting the need for new diagnostic and therapeutic strategies; therefore, this study integrates genomic, transcriptomic, single-cell, methylomic, and molecular-dynamics data to pinpoint pathogenic variants. We performed an integrative multi-omics analysis of publicly available datasets. Somatic variants from 23 tumor samples in The Cancer Genome Atlas were annotated with 11 pathogenicity tools (AUC ≥ 0.86 across EVE, REVEL, SIFT, AlphaMissense, DEOGEN2 were the most stringent). Differential gene expression was assessed in matched bulk RNA-seq (tumor vs. non-tumor) using DESeq2 with Benjamini–Hochberg FDR correction. A single-cell RNA-seq dataset comprising 23,782 cells from an intrahepatic cholangiocarcinoma was clustered with marker genes identified by Wilcoxon rank-sum tests. Illumina 450 K methylation arrays (52 tumors, 12 normal livers) were analyzed with limma and DMRcate to detect differentially methylated probes and regions. AlphaFold3 models of wild-type and MAP2K1^*R*49*C*^ were subjected to 50 ns all-atom molecular-dynamics simulations in GROMACS; conformational shifts were quantified by RMSD/RMSF and stability tested with FoldX5. Twenty-four tumor-specific missense variants were detected. The four highest-confidence pathogenic substitutions (EVE, REVEL, SIFT, AlphaMissense, DEOGEN2) occurred in *TUBB3*, *FLNC*, *ABCA1*, and *MAP2K1*. Bulk RNA-seq confirmed significant dysregulation of these genes and enrichment of extracellular-matrix organization, cytoskeletal remodeling, MAPK signaling, and cholesterol-efflux pathways. Single-cell analysis resolved 23 transcriptionally distinct clusters; proliferative malignant cholangiocytes selectively over-expressed *ABCA1* and *MAP2K1*, indicating tumor-cell specificity. Methylome profiling identified 148,928 DMPs and 7040 DMRs; promoter hypomethylation of *TUBB3* and *ABCA1* correlated with their transcriptional activation. Substituting Arg-49 with Cys in MAP2K1 dismantles the Arg-centred hydrogen-bond/salt-bridge cluster, reduces hydrophobic packing, and, corroborated by 50 ns MD (Welch’s t = −58.06, *p* = 3.17 × 10^−230^) and FoldX5 (ΔΔG = +2.3 kcal mol^−1^), significantly destabilises the protein, manifesting as higher backbone RMSD and increased local flexibility relative to wild type. This multi-omics, public data-driven synthesis delineates a coherent network of genomic, epigenomic, transcriptomic, and structural vulnerabilities, offering a rational framework for therapeutic targeting of cholangiocarcinoma. This study reveals novel bile duct-associated variations that expand our understanding of cholangiocarcinoma pathogenesis and provide potential targets for precision medicine approaches.

## 1. Introduction

Bile duct cancer, also known as cholangiocarcinoma (CCAs), is a rare and highly lethal malignancy of epithelial cells that can arise at any point along the biliary tree or within the liver parenchyma [1]. Comprehensive genomic and epigenomic research has revealed that the molecular landscapes of CCAs vary significantly depending on their etiology, emphasizing that both extrinsic and intrinsic carcinogenic pathways can form multiple cancer subtypes within the same organ [2]. The CCA affects approximately 8000 individuals in the United States annually. According to world statistics, the 5-year relative survival rate for patients with bile duct cancer is 21.7%, which indicates that the prognosis for these individuals is still unfavorable [3]. Based on the Global Cancer Observatory, the age-standardized incidence rate for liver and intrahepatic CCA (iCCA) in the world was 8.6 per 100,000 individuals in 2022 [4].

The etiological risk factors for CCA include primary sclerosing cholangitis, certain parasitic infections such as *Clonorchis sinensis* and *Opisthorchis viverrini*, and hepatolithiasis. Despite these associations, a definitive etiological factor remains unclear in most cases. CCAs predominantly occur in elderly populations, with the majority of patients being 60 years or older [5]. Given the complex and poorly understood etiology of this malignancy, there is a critical need to identify and functionally characterize the genetic alterations that drive its development [6].

This study aims to elucidate the functional relevance of somatic variations in CCA by integrating genomic, transcriptomic, single-cell, and genome-wide DNA methylation and atomistic molecular-dynamics (MD) simulations. Since CCA is a rare form of cancer and considered an orphan cancer in terms of research, our goal is to predict the pathogenicity of variants, using with the TCGA database and additional datasets. We compiled an extensive set of candidate variants in CCA, establishing a foundation for systematic validation and functional interrogation through bulk- and single-cell transcriptomics, genome-wide methylation profiling, and molecular-dynamics simulations.

## 2. Results

**Figure 1:**
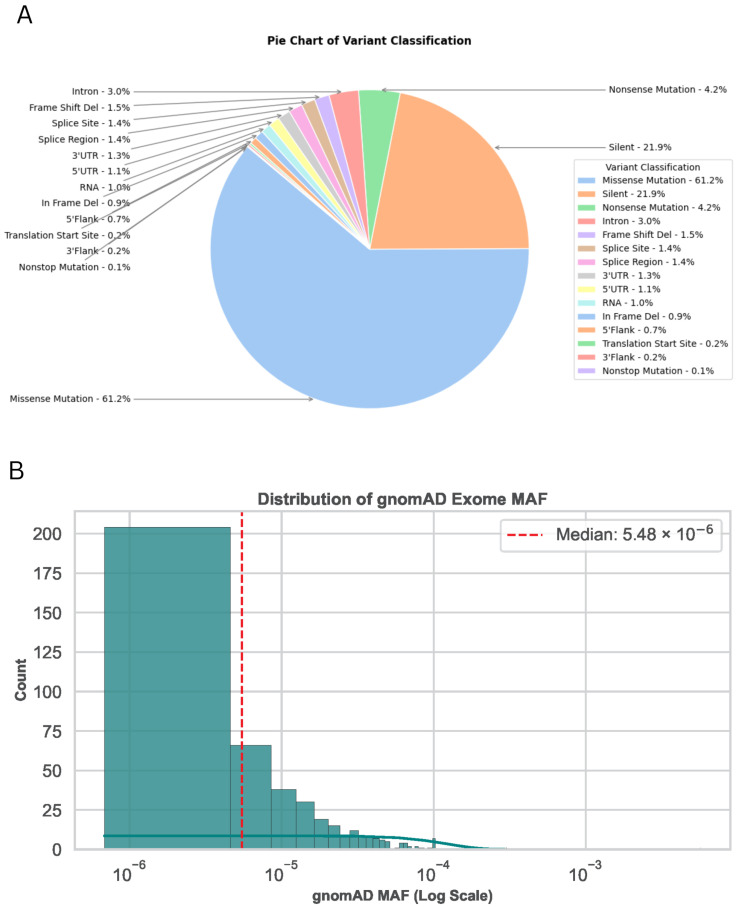
Somatic variant analysis of 1017 variations in 23 tumor samples. This figure demonstrates the classification distribution and the population allele frequency distribution from gnomAD exome data. (**A**) Pie chart of variant classification. The majority of the variants are missense mutations (61.2%), followed by silent mutations (21.9%) and nonsense mutations (4.2%). Other variant types, including intronic, splice site, and frameshift mutations, contribute to the remaining proportion. (**B**) Histogram of gnomAD MAF distribution on a log scale, showing the frequency of observed somatic variants in the population. The red dashed line indicates the median MAF value (5.48 × 10^−6^), highlighting the rarity of most variants in the general population.

Somatic variation profiling of 23 tumors revealed 1017 alterations dominated by missense mutations (61.2 %), while gnomAD exome [7] data showed these variants are exceedingly rare in the general population (median MAF about 5.5 × $10^{-6}$), indicating a prevalently protein-altering and tumor-specific mutational landscape (Figure 1).

In variation pathogenicity prediction annotations, notably, EVE (Evolutionary Model of Variant Effect) (AUC = 1.00), REVEL (Rare Exome Variant Ensemble Learner) (AUC = 0.95), SIFT (Sorting Intolerant From Tolerant) (AUC = 0.93), AlphaMissense (AUC = 0.86) and DEOGEN2 (Deleteriousness Prediction of Genetic Variants 2) (AUC = 0.91) scores emerged as the top-performing predictors, with pathogenicity thresholds above 0.5. Of the 24 identified variants, six are classified as pathogenic in ClinVar, whereas 18 are VUS or remain unannotated (Supplementary Table S1).

The TCGA database suggested 61 cancer-related variants. Of these, 9 variants were found to overlap with our set of 24 variations, which were predicted as pathogenic, occurring in key cancer-associated genes such as *SMAD4*, *IDH1*, *KRAS* (at two distinct loci), *TP53* (at three distinct loci), *PTCH1*, and *MAP2K1*. The recurrence of variations in pivotal genes like *TP53* and *KRAS*, both of which play central roles as tumor suppressors and oncogenes, respectively, supports the biological relevance of the identified alterations and validates the robustness of the variant prioritization strategy implemented in this study.

Across the 11 pathogenicity-prediction tools assessed on cholangiocarcinoma variants, performance fell into three tiers: EVE led with a flawless AUC of 1.0 and balanced operating points (Accuracy = 0.85, Recall = 0.82, F1 = 0.90); a second cluster, MetaRNN, VEST4, SIFT, and DEOGEN2, shared near-identical radar-plot footprints, combining perfect Precision with high Accuracy (0.87–0.90) and strong Recall (0.83–0.86), yielding F1 scores > 0.91; AlphaMissense trailed slightly, exchanging a small Recall dip for an F1 of 0.88. In contrast, REVEL posted an excellent AUC (0.95) but low Recall (0.57), risking false negatives, whereas CADD showed the inverse (low AUC 0.64, high Recall 0.89) and MetaLR and M-CAP lagged on both sensitivity and specificity (Table 1). Mechanistically, these tools tap four complementary evidence streams—evolutionary conservation, variant-rarity cues, 3-D structure context, and meta-ensemble aggregation. EVE and SIFT anchor the conservation axis, quantifying intolerance to change from deep multiple-sequence alignments [8,9]; DEOGEN2 layers conservation with allele-frequency rarity, gene-intolerance metrics, and regional annotations [10]; REVEL acts as a stacked meta-ensemble synthesizing outputs from a dozen algorithms spanning biochemical, population, and conservation features and trained on rare neutral variants (allele-frequency < 0.5 %) [11]; and AlphaMissense enriches predictions with explicit structural insight by embedding AlphaFold-predicted environments into a large language model [12]. Because EVE, SIFT, DEOGEN2, REVEL, and AlphaMissense collectively cover all four signal streams while ranking among the top performers, they constitute a balanced, orthogonal filter set for confidently prioritizing pathogenic bile-duct-cancer variants.

**Figure 2:**
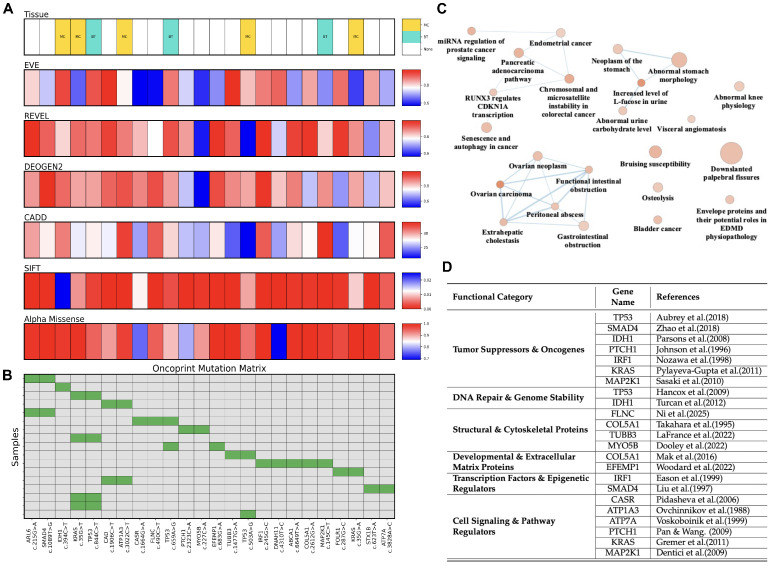
OncoPrint of Somatic Variations in TCGA CCA Cohort. (**A**) Bar indicates the tissue origin of each sample, with labels denoting “BT” (biliary tract), “MC” (multiple cancer types), and “None” where tissue annotation is missing. Below, multiple tracks represent variant pathogenicity and functional impact scores from different computational tools: EVE, REVEL, DEOGEN2, CADD, SIFT, and AlphaMissense, shown as continuous heatmaps. Red color typically denotes higher predicted pathogenicity, while blue shades represent lower impact scores. Each column corresponds to a single sample and variation. (**B**) Labeled “Mutation Matrix” shows a binary matrix of nonsynonymous somatic variations in samples. Each green tile denotes the presence of a specific variant (e.g., c.123A>T) in a given gene across the patient cohort, while gray indicates absence. (**C**) Functional categorization of 24 variant-associated genes, grouped into key biological roles (**D**) Overrepresentation enrichment analysis of these genes in WikiPathways and Human Phenotype Ontologies, visualized using g:Profiler, highlighting enriched biological processes and pathways [13,14,15,16,17,18,19,20,21,22,23,24,25,26,27,28,29,30,31,32,33,34,35].

In Figure 2A, an integrated oncoprint visualization of somatic variations observed in TCGA CCA cohort is presented. Most variations originate from the biliary tract (BT, cyan), with multiple cancer types (MC, yellow) and several unannotated variations (white). Across six independent in-silico predictors (EVE, REVEL, DEOGEN2, CADD, SIFT, AlphaMissense), the majority of substitutions score in the high-impact range (deep red), indicating a strong consensus for pathogenicity. Figure 2B maps these variations across individual samples and highlights recurrent hotspots: *ARL6* c.215G>A in two patients, *SMAD4* c.1089T>G in two, *KRAS* c.35G>T in four, *TP53* c.844C>T in four, *CAD* c.1906C>T in two, *ATP1A3* c.3022C>T in two, and the additional *TP53* variants c.659A>G and c.503A>G, each present in two patients. The remaining variants are singletons, each identified in one patient.

Several enriched terms are associated with bile duct cancer, including the pancreatic adenocarcinoma pathway, functional abnormality of the gastrointestinal tract, extrahepatic cholestasis, and peritoneal abscess, suggesting the involvement of CCA-associated genes in biliary tract dysfunction, oncogenic signaling, and tumor development (Figure 2C). 24 variant-associated genes are grouped in key biological roles including tumor suppressors & oncogenes, DNA repair & genome stability, structural & cytoskeletal proteins, developmental & extracellular matrix proteins, transcription factors & epigenetic regulators, and cell signaling & pathway regulators (Figure 2D).

**Figure 3:**
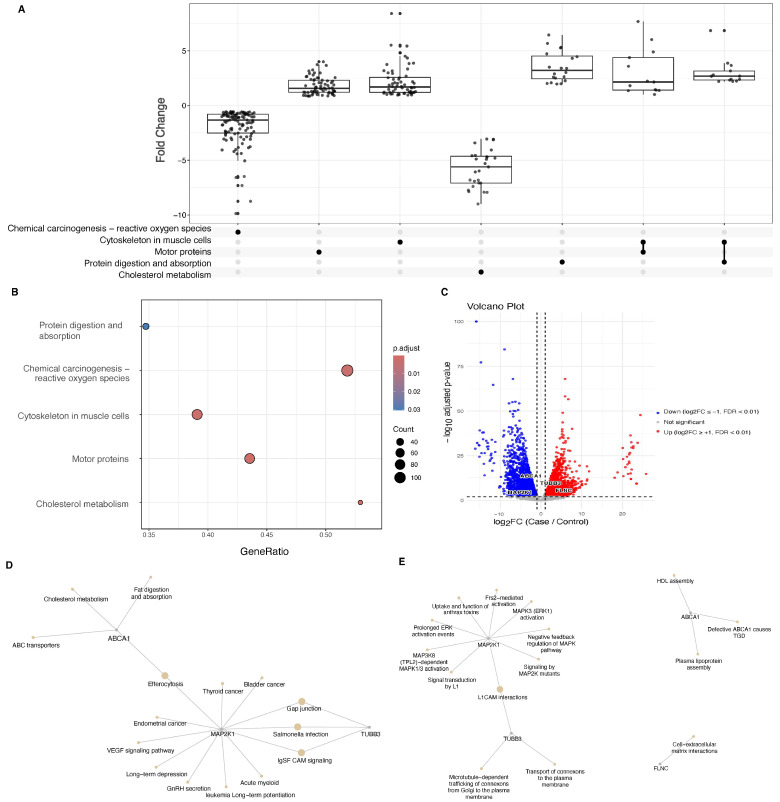
Transcriptomic and pathway analysis of differentially expressed genes (DEGs) in bile duct tumors from the TCGA and GSE63420 dataset. (**A**)The UpSet plot illustrates the intersection of significantly enriched KEGG pathways derived from the differentially expressed genes (DEGs) in bile duct tumors. Each box plot represents the distribution of gene expression fold changes for genes involved in a specific pathway such as those in chemical carcinogenesis, cytoskeleton organization, motor proteins, and cholesterol metabolism. (**B**) Dot plot of KEGG pathway enrichment analysis, where the x-axis represents the gene ratio, the size of the bubbles indicates the number of genes involved, and the color gradient represents the adjusted *p*-value. (**C**) Volcano plot illustrating the distribution of DEGs, with significantly upregulated genes shown in red, downregulated genes in blue, and non-significant genes in gray. Key DEGs, including *TUBB3*, *FLNC*, *MAP2K1*, and *ABCA1*, are highlighted due to their involvement in enriched pathways related to bile duct tumorigenesis. (**D**) KEGG gene–pathway network centered on *ABCA1*, *TUBB3*, *FLNC*, and *MAP2K1*, linking these genes to cholesterol metabolism/ABC transporters, gap junctions, cytoskeletal regulation, and MAPK signaling; gray nodes denote genes and yellow nodes denote enriched KEGG pathways. (**E**) Reactome gene–pathway network for the same four genes, connecting *ABCA1* to HDL assembly and lipid efflux, *MAP2K1* to the RAF/MAPK cascade, TUBB3 to gap-junction/connexin trafficking and microtubule processes, and *FLNC* to L1CAM interactions and actin cytoskeleton organization; gray nodes denote genes and yellow nodes denote enriched Reactome pathways.

In Figure 3, differential-expression analysis of the GSE63420 iCCA cohort identified four genes, in 24 variant-associated genes, displaying both strong statistical support (adjusted *p* value (padj) < 1 × $10^{-8}$) and substantial fold changes. *MAP2K1* was significantly down-regulated ($\log_{2}\mathrm{FC}$ = −1.57, padj = 1.85 × $10^{-7}$), corresponding to approximately 2.6-fold elevation over controls, consistent with aberrant MAPK signaling in biliary tumorigenesis. In contrast, *TUBB3* ($\log_{2}\mathrm{FC}$ = 3.26, padj = 8.0 × $10^{-14}$), respectively—despite high basal expression, highlighting cytoskeletal reprogramming and extracellular-matrix remodeling in the malignant epithelium. Genes annotated to Cytoskeleton in muscle cells, Motor proteins, and Protein digestion & absorption are predominantly up-regulated, whereas Cholesterol metabolism and Chemical carcinogenesis—reactive oxygen species are largely down-regulated. The UpSet matix beneath the boxplots highlights gene sharing, with prominent intersections between Cytoskeleton in muscle cells and Motor proteins, while the down-regulated cholesterol and ROS modules contribute few shared genes with the other pathways. (Figure 3A,B). *FLNC* showed a similar up-trend ($\log_{2}\mathrm{FC}$ = 2.99, padj = 1.2 × $10^{-7}$) from a lower baseline, reinforcing the disruption of actin-binding networks. Conversely, *ABCA1* was markedly reduced ($\log_{2}\mathrm{FC}$ = −3.41, padj = 2.78 × $10^{-13}$), implicating cholesterol efflux dysregulation in tumor metabolism (Figure 3C). All differential expression statistics are shown in Supplementary Table S2. Importantly, GSEA analysis further revealed that these genes converge on significant pathways, including chemical carcinogenesis–reactive oxygen species, cytoskeletal regulation, motor protein function, protein digestion and absorption, and cholesterol metabolism, all of which are strongly associated with bile duct cancer. In CCA, pathway mapping shows *ABCA1* (cholesterol/ABC transport) and *MAP2K1* (RAF–MEK–ERK) are downregulated, while *TUBB3* (gap-junction/connexin trafficking, microtubules) and *FLNC* (actin–focal-adhesion modules) are upregulated. This configuration indicates reduced cholesterol efflux and transcriptional attenuation of MEK signaling alongside heightened cytoskeletal and junctional remodeling, converging to rewire RTK/MAPK–membrane microdomain crosstalk and favor invasive phenotypes (Figure 3D,E).

**Figure 4:**
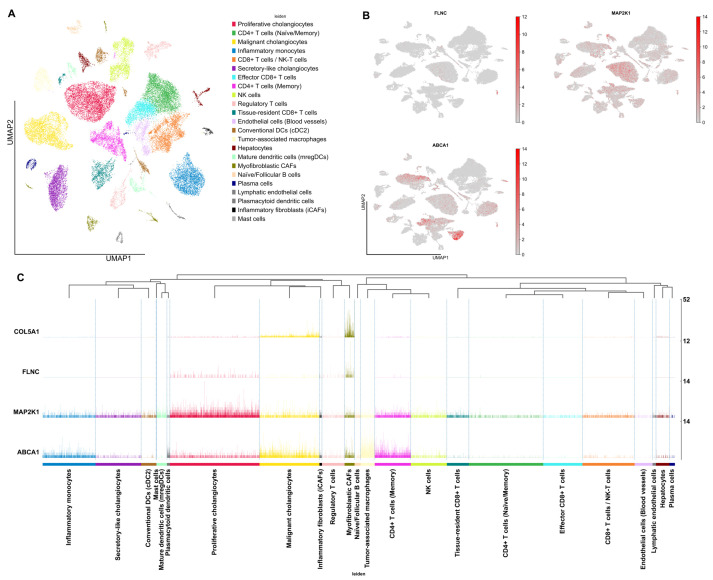
UMAP visualization of single-cell transcriptomes from iCCA and adjacent tissues. (**A**) Cells are colored by Leiden clusters and annotated based on cell type identities inferred from marker gene expression and known lineage markers. A total of 23 transcriptionally distinct clusters were identified, representing a diverse array of cell types including cholangiocyte subpopulations (proliferative, malignant, and secretory-like), immune cells (CD4+ and CD8+ T cells, NK cells, dendritic cells, macrophages), stromal cells (cancer-associated fibroblasts), endothelial cells, and hepatocytes. This map highlights the cellular heterogeneity within the iCCA tumor microenvironment and surrounding tissue. (**B**) UMAP plots display the expression distribution of each gene across the cellular landscape. Each point represents an individual cell, with red intensity indicating higher expression levels. These plots highlight cell-type-specific expression patterns and potential spatial heterogeneity within the tumor microenvironment. (**C**) A hierarchical clustering heatmap shows gene expression intensity (y-axis) across annotated cell populations (x-axis).

Single-cell RNA-seq UMAP embedding resolved 23 transcriptionally discrete clusters—including proliferative, malignant, and secretory-like cholangiocytes alongside diverse immune, stromal, endothelial, and parenchymal populations—thereby underscoring the marked cellular heterogeneity characterizing the iCCA microenvironment and its surrounding tissue (Figure 4A).

Single-cell RNA-seq mapping of *FLNC*, *MAP2K1*, and *ABCA1* reveals highly specific, lineage-restricted transcriptional programs within the bile duct tumor microenvironment. *FLNC* transcripts are predominantly localized to myofibroblastic cancer-associated fibroblast (CAF) clusters ($\log_{2}\mathrm{FC}$: 4.35, padj = 1.88 × 10^−63^; $\log_{2}\mathrm{FC}$: 4.58, padj = 9.99 × 10^−5^), consistent with their respective roles in extracellular matrix organization and cytoskeletal remodeling. Statistically, *FLNC* shows moderate upregulation ($\log_{2}\mathrm{FC}$: 2.30, padj = 0.039). In contrast, *MAP2K1* is broadly expressed across cholangiocyte lineages but is most enriched in the malignant and highly proliferative subsets ($\log_{2}\mathrm{FC}$: 25.16, padj = 8.29 × 10^−139^), aligning with MAPK pathway activation in tumor cells. *ABCA1* is strongly expressed in both malignant cholangiocytes ($\log_{2}\mathrm{FC}$: 23.16; padj = 1.35 × 10^−117^) and tumor-associated macrophages ($\log_{2}\mathrm{FC}$: 4.09; padj = 2.45 × 10^−279^), suggesting dysregulated cholesterol efflux in both epithelial and immune compartments. Additionally, *ABCA1* shows moderate expression in proliferative cholangiocytes ($\log_{2}\mathrm{FC}$: 1.83, padj = 0.115), although statistical significance is marginal. These transcriptional patterns are corroborated by a hierarchical heatmap, which clusters *FLNC* with fibroblast populations, *MAP2K1* with epithelial compartments—particularly the proliferative subset—and *ABCA1* at the interface of epithelial and myeloid branches (Figure 4B,C).

Genome-wide methylation profiling of 449,351 CpG dinucleotides revealed widespread epigenetic remodeling in iCCA, with 148,375 sites (33%) exceeding the false discovery rate (FDR)-adjusted significance threshold of 0.05. Gene-centric analyses highlight four cancer-relevant loci *MAP2K1*, *FLNC*, *ABCA1*, and *TUBB3* as key targets of methylation deregulation, each exhibiting distinct CpG methylation patterns linked to their known biological roles in tumorigenesis.

At the *MAP2K1* locus on chromosome 15, focal hypomethylation is observed at several CpGs. This configuration may modulate MAPK signaling through context-dependent feedback regulation. Notably, *MAP2K1* is downregulated in bulk RNA-seq data, and its methylation metrics (padj = 0.0463, normalized enrichment score (NES) = −1.779) indicate a negative correlation that deviates from the classical methylation-expression model. This suggests that hypomethylation at this locus may contribute to repression of *MAP2K1* expression in a context-dependent manner, potentially altering feedback inhibition and sustaining oncogenic signaling.

*FLNC* (chr 7q32.1) is upregulated in RNA-seq, and methylation statistics (padj = 0.0278, NES = 1.589) suggest that hypermethylation in the gene body may be associated with increased transcription. This observation diverges from the classical promoter methylation-expression model but is consistent with reports that gene body hypermethylation can enhance transcriptional elongation and stability, thereby contributing to elevated *FLNC* expression in this context.

Interestingly, *TUBB3* (chr 16q24.3) is upregulated in bulk RNA-seq, and the methylation metrics (padj = 0.3912, NES = −1.233) suggest a negative correlation, consistent with the canonical methylation-expression model in which hypomethylation is associated with transcriptional activation.

Lastly, *ABCA1* (chr 9q31.1) exhibits a biphasic methylation shift, with hypomethylation upstream and hypermethylation downstream, possibly disrupting enhancer–promoter communication. It demonstrates pronounced hypermethylation along its CpG-island shore. Consistently, *ABCA1* is downregulated in RNA-seq, with methylation metrics (padj = 0.2446, NES = 1.320), fitting the canonical model in which promoter and shore hypermethylation contribute to transcriptional repression.

**Figure 5:**
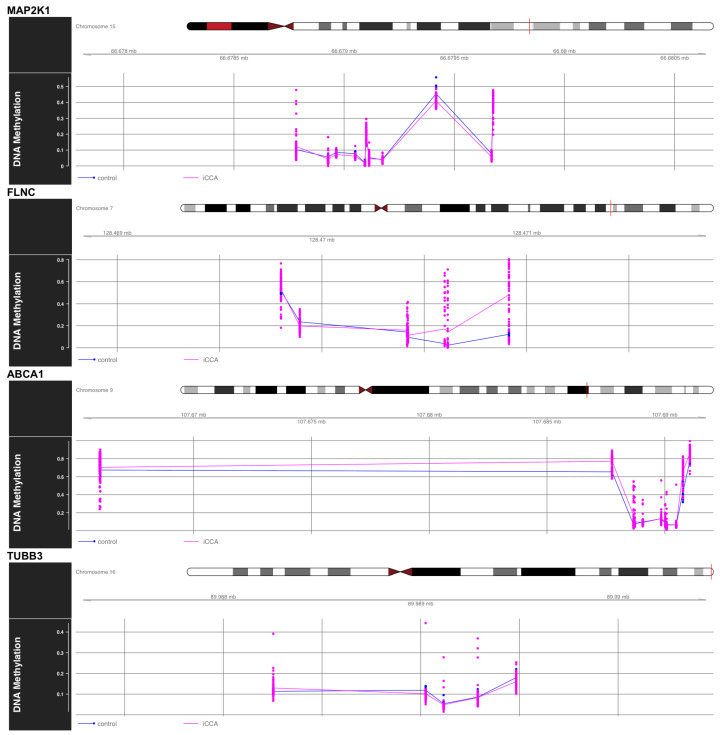
Differential DNA methylation patterns in selected genes associated with iCCA. DNA methylation profiles for four candidate genes *MAP2K1*, *FLNC*, *ABCA1*, and *TUBB3* are shown with comparisons between control (blue) and iCCA (magenta) samples. Each panel represents one gene locus along its corresponding chromosome, with CpG methylation beta values plotted vertically and genomic coordinates shown horizontally.

These data illustrate how iCCA exhibits both hyper- and hypomethylation events that converge on genes regulating signaling (*MAP2K1*), extracellular matrix dynamics (*FLNC*), lipid metabolism (*ABCA1*), and cytoskeletal structure (*TUBB3*). The methylation-expression model is variably upheld across these genes, underscoring the gene- and context-specific nature of epigenetic regulation in cholangiocarcinoma Figure 5.

50-ns atomistic molecular-dynamics simulations carried out in GROMACS compared wild-type MAP2K1 with the p.R49C variant, revealing that the variant increases conformational change, which could enhance aberrant MEK1 signaling and thereby promote oncogenic activity [36].

**Figure 6:**
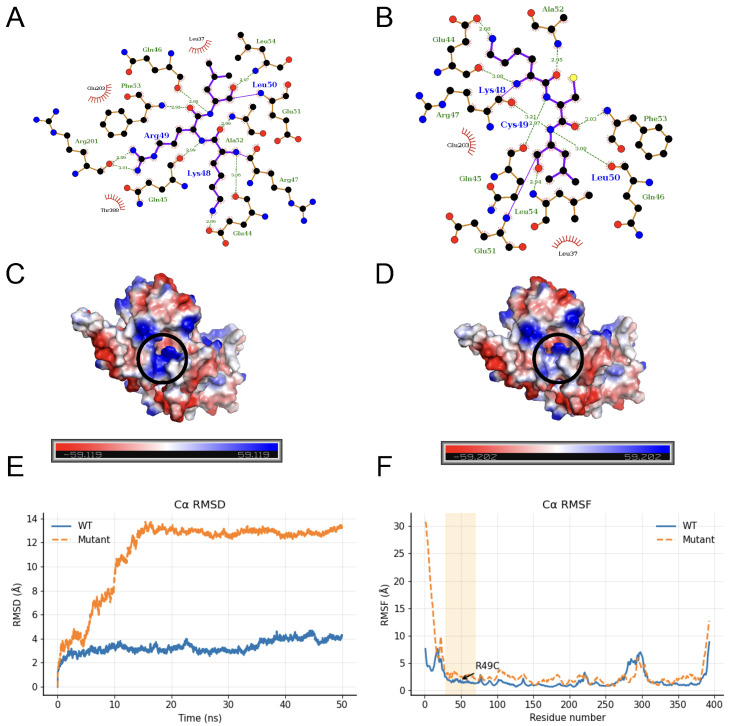
Structural and dynamic effects of the MAP2K1 p.R49C variant (**A**,**B**) 2-dimensional interaction diagrams of the (**A**) MAP2K1 wild-type (WT) protein structure and (**B**) its p.R49C variant show differences in hydrogen bonding and hydrophobic contacts within the active site. The WT Arg49 forms several stabilizing interactions, which are lost or altered in the mutant due to substitution with cysteine. (**C**,**D**) (**C**) Electrostatic surface potential maps of WT and (**D**) mutant MAP2K1 protein represent changes in charge distribution around the mutation site (black circle), potentially affecting local interactions and binding dynamics. (**E**) RMSD plot of backbone Cα atoms over 50 ns molecular dynamics simulation, indicating higher structural fluctuations in the mutant (orange dashed line) compared to WT (blue solid line). (**F**) Per-residue Cα RMSF profiles show that the p.R49C variant (orange, dashed) exhibits markedly greater flexibility than the wild type (blue, solid) within the N-terminal window surrounding the mutation (residues 39–59, shaded), underscoring a localized destabilization centred on residue 49 (arrow).

The LigPlot++ contact diagrams reveal that substituting Arg-49 with Cys markedly thins and rewires the local interaction network. In the wild-type map (Figure 6A) the guanidinium of Arg49 behaves as a polar hub: it donates two short hydrogen bonds (<3.0 Å) to the main-chain carbonyls of Lys48 and Ala52, accepts a third from the Leu50 backbone (2.97 Å), and engages in a salt-bridge-like contact with Glu44, while its long aliphatic arm packs against Phe53 and Gln46. These electrostatic and hydrophobic anchors are lost in the mutant (Figure 6B). The smaller, uncharged Cys49 forms only two longer H-bonds (3.21 Å to Lys48 and 2.97 Å to the Leu50 carbonyl) and lacks any ionic partner; the void is partially filled by a new Cys49–Leu54 van-der-Waals contact, but the overall density of polar links and hydrophobic interaction drops. Consequently, the mutant network is less cross-linked: the Arg-mediated electrostatic triad is lost, its interactions become more diffuse, and the resulting structural changes align with the destabilizing ΔΔG and elevated RMSD observed in our MD and FoldX analyses (Figure 6A,B). Aligning with these findings, electrostatic maps indicate a local redistribution of charge around the mutation (black-circled region), a change that could plausibly contribute to weakening favorable contacts (Figure 6C,D). Over the 50 ns molecular-dynamics run, the mutant’s backbone Cα RMSD climbs more rapidly and attains higher values than the wild-type, reflecting greater global conformational drift, while the per-residue RMSF profile shows amplified flexibility at N-terminus and around the mutation site. Block-averaging produced 45 independent RMSD blocks for each system (WT and mutant), and both Welch’s *t*-test (t = −58.06, *p* = 3.16 × 10^−230^) and the Kolmogorov–Smirnov test (D = 0.911, *p* = 1.89 × 10^−220^) demonstrate that the mutant’s RMSD distribution is significantly different from—and markedly higher than—the wild type (Figure 6E,F). Consistent with these observations, FoldX ΔΔG analysis averaged over 20 independent replicas predicted a positive stability change of +2.303 kcal mol^−1^, confirming that the p.R49C substitution is energetically destabilizing relative to the wild-type.

Together, these findings suggest that the R49C substitution perturbs the active-site network, alters surface electrostatics, and increases overall and local mobility, which could impair structural integrity and functional interactions of MAP2K1.

## 3. Discussion

To comprehensively characterize the molecular landscape of CCA, we implemented an integrative multi-omics workflow combining somatic variant annotation, bulk and single-cell transcriptomics, methylation profiling, and molecular dynamics simulations. Somatic variations from 23 TCGA bile duct tumor samples were annotated and evaluated with 11 in silico pathogenicity tools; EVE, REVEL, SIFT, AlphaMissense, and DEOGEN2 were selected based on superior predictive performance (AUC ≥ 0.86) (Table 1). Variants classified as pathogenic across all five tools were cross-referenced with known cancer genes, identifying high-confidence mutations in *FLNC*, *MAP2K1*, and *ABCA1*. To evaluate transcriptional impact, we integrated bulk RNA-seq data (GSE63420), identifying these genes as significantly differentially expressed in tumor versus healthy tissues (Figure 3). Further, scRNA-seq analysis using the GSE138709 dataset (processed in scanpy) revealed cell-type-specific expression of these genes across 23 transcriptionally distinct clusters. UMAP projections and hierarchical clustering showed *FLNC* enriched in fibroblast-like and stromal clusters, while *MAP2K1* and *ABCA1* were predominantly expressed in malignant epithelial populations (Figure 4). We prioritized *MAP2K1*, *TUBB3*, *FLNC*, and *ABCA1* because they harbored high-confidence, tumor-specific variants that passed our multi-tool pathogenicity prioritization and were among the most significantly dysregulated genes in the bulk transcriptome (with scRNA-seq support), nominating them as convergent genomic–transcriptional candidates for follow-up.

To assess epigenetic regulation, we analyzed DNA methylation data from iCCA samples (GSE201241) using the minfi and limma pipelines. Methylation profiling revealed gene-specific patterns that both conform to and deviate from classical models: *MAP2K1* showed focal hypomethylation yet remained downregulated, suggesting context-dependent feedback regulation; *FLNC* exhibited gene body hypermethylation correlating with increased transcription; *TUBB3* displayed canonical hypomethylation-activation correlation; and *ABCA1* demonstrated biphasic methylation with CpG-shore hypermethylation linked to transcriptional repression, underscoring gene-specific epigenetic regulation in cholangiocarcinoma (Figure 5).

Finally, to assess the structural impact of the variation, we performed MD simulations on the wild-type and mutant (p.R49C) MAP2K1 protein using AlphaFold3 models and GROMACS. The mutant displayed increased structural fluctuation, indicating potential conformational instability that may influence kinase activity and contribute to oncogenic signaling (Figure 6). Overall, this integrative pipeline links genetic variation, transcriptional dysregulation, epigenetic remodeling, and protein structural perturbation, providing a robust functional framework for identifying and characterizing novel driver events in CCA.

Applying the full multi-omics workflow to all recurrent, pathogenic variants highlighted *MAP2K1*, *FLNC*, *TUBB3*, and *ABCA1* as the most compelling driver candidates; we therefore integrate our structural, epigenetic and transcriptomic findings with the mechanistic and clinical literature for each gene to delineate their individual and collective contributions to CCA pathogenesis.

*TUBB3* encodes class III β-tubulin, a microtubule subunit normally found in neurons but also aberrantly expressed in various cancers. Proteomic studies have identified TUBB3 as a distinguishing marker in CCA. In this study, researchers found that βIII tubulin was highly expressed in approximately half of iCCA, especially the peripheral (small-duct) subtype, while it was largely absent in hepatocellular carcinomas and benign bile duct lesions. Immunohistochemical validation showed *TUBB3* positivity in 50% of peripheral CCA versus only 15% of perihilar tumors [37]. While such expression may reflect microtubule reprogramming and support diagnostic/stratification use, its CCA-specific predictive utility for taxane benefit remains unproven and requires prospective validation [38]. Indeed, TUBB3 (along with neural cell adhesion molecule, NCAM) tends to label *small*-duct type CCA, albeit with lower sensitivity than markers like N-cadherin or C-reactive protein [39].

Bile duct cancers are known for a desmoplastic stroma—a rich collagenous matrix produced by cancer-associated fibroblasts (CAFs) that surrounds tumor cells [40]. Type V collagen, though a minor collagen, regulates fiber assembly and tissue stiffness, influencing how tumor cells interact with their microenvironment [41].

While direct studies on *FLNC* in CCA are limited, related family member filamin A (*FLNA*) has been implicated in iCCA progression. *FLNC* was identified as upregulated in iCCA relative to normal liver, suggesting a potential involvement in tumorigenesis and progression; subsequent studies confirmed its overexpression in CCA, associating it with adverse prognosis and modified tumor microenvironment attributes [42]. High *FLNA* expression (and its active 90 kDa fragment from calpain-mediated cleavage) correlates with more aggressive disease, and blocking FLNA cleavage reduced iCCA cell proliferation and migration [43]. This finding suggests that filamin-mediated cytoskeletal reorganization can drive CCA aggressiveness. Given FLNC’s similar actin-binding function, its dysregulation may likewise influence tumor cell invasion and metastasis. The *FLNC* signal aligns with the desmoplastic stroma of iCCA—where myofibroblastic CAF programs shape invasion, immune exclusion, and therapeutic resistance—and is supported by related filamin biology in iCCA; translating stromal biology into clinical benefit will likely require rational combinations and biomarker-anchored selection [40,43].

Studies in other tumor types illustrate *FLNC*’s potential dual role in cancer. For example, in gastric and prostate cancers, *FLNC* was associated with increased invasiveness–*FLNC* knockdown enhanced cell migration/invasion, whereas restoring *FLNC* curbed these malignant behaviors [44]. Also, upregulation of *FLNC* has been observed in advanced liver tumors: a proteomics study in hepatocellular carcinoma found *FLNC* levels rose with tumor stage and contributed to invasiveness. These findings imply that the impact of *FLNC* is context-dependent but clearly tied to cell motility [45].

*ABCA1* is a membrane transporter that exports cholesterol and phospholipids from cells to apolipoproteins (forming high-density lipoprotein (HDL) particles). CCA cells appear to benefit from cholesterol dysregulation. A study by Seeree et al. demonstrated that disturbances in cholesterol homeostasis (transport interruption and intracellular cholesterol accumulation) can promote CCA initiation and progression. In CCA cell lines, *ABCA1* (and its partner transporter *ABCG1*) are expressed and actively mediate cholesterol efflux to HDL. Treatment with statins (cholesterol-lowering drugs) like simvastatin or atorvastatin had anti-proliferative effects on these CCA cells, correlating with reduced intracellular lipid levels and downregulation of *ABCA1*/*ABCG1* expression. This paradoxical downregulation (statins reduce cholesterol synthesis, which in turn can suppress ABCA1 expression) was associated with decreased cell viability. However, when cells were pre-loaded with excess cholesterol, the statins’ efficacy dropped, and *ABCA1*/*ABCG1* function persisted despite drug treatment [46]. This suggests that a cholesterol-rich environment (such as high patient dietary cholesterol or tumor stroma resources) can undermine cholesterol-targeting therapies by sustaining the cholesterol efflux needed for tumor survival.

*MAP2K1* encodes MEK1, a dual-specificity kinase that is a central component of the RAS/RAF/MEK/ERK (MAPK) signaling cascade. This pathway regulates cell proliferation, survival, and differentiation, and is frequently hyperactivated in cancers. Aberrant MAPK signaling is a well-documented feature of CCA. Though *MAP2K1* variations are relatively rare in these tumors, occurring in a small subset of cases (e.g., 4% co-occurring with *IDH1* mutations in one study) [47], the pathway is often activated by upstream alterations. For instance, *KRAS*, the upstream RAS GTPase, is mutated in approximately 20–25% of CCA, especially in extrahepatic and large-duct subtypes. These *KRAS* mutations constitutively fire the RAF-MEK-ERK cascade. Additionally, receptor tyrosine kinase fusions (FGFR2 fusions in intrahepatic CCA) or variations (EGFR, BRAF, etc.) can also funnel into MEK/ERK activation [48]. With respect to the MAPK axis, population-level genomics place frequent activation via upstream lesions (e.g., *KRAS*) across biliary tract cancers, reinforcing the centrality of RAS–RAF–MEK–ERK signaling. Yet, in unselected CCA populations, MEK-pathway suppression has not changed standard care: adding selumetinib to cisplatin/gemcitabine failed to improve efficacy and increased toxicity [49], and atezolizumab plus cobimetinib showed only modest progression-free survival gains with low response rates versus atezolizumab alone [50], while an immunotherapy triplet variation did not improve outcomes [51]. Against this backdrop, our structural/molecular-dynamics analyses indicate that *MAP2K1* (MEK1) p.R49C perturbs stabilizing interactions and protein dynamics, plausibly altering pathway output; however, actionable sensitivity to MEK/ERK inhibitors is likely to be mutation-class–dependent and remains sparsely characterized specifically in CCA, arguing that MEK/ERK-directed strategies in *MAP2K1*-altered or MAPK-activated iCCA should at present be prioritized within biomarker-selected clinical trials rather than routine off-label use.

Our findings should be interpreted within the rapidly evolving precision-oncology landscape of cholangiocarcinoma, in which actionable alterations—most prominently *FGFR2* fusions and *IDH1* variations in intrahepatic disease—already support targeted options with prospective evidence (e.g., futibatinib in *FGFR2*-rearranged iCCA; ivosidenib in *IDH1*-mutant CCA). These benchmarks provide a frame of reference for rarer events prioritized here [52].

## 4. Materials and Methods

### 4.1. Data Collection and Variant Extraction

Bile duct cancer mutation data were acquired from TCGA Genomic Data Commons (GDC) portal. A total of 23 tumor samples and 23 matched healthy tissue samples were analyzed, with somatic mutation data extracted from Mutation Annotation Format (MAF) files [53]. Only variants present in tumor tissues were retained for further analysis. This ensured that the identified variations were specifically associated with cancerous transformations rather than normal genetic variation.

### 4.2. Variation Annotation

The identified tumor-specific variations were annotated using ANNOVAR (accessed on 29 July 2025) URL: https://annovar.openbioinformatics.org/en/latest/, incorporating ClinVar data to classify pathogenicity [54]. To enhance annotation accuracy, multiple computational predictive tools were used, including M-CAP, MetaRNN, VEST4, CADD, REVEL, AlphaMissense, EVE, SIFT, Polyphen, DEOGEN2, and MetaLR. These tools assess the potential pathogenicity of mutations based on evolutionary conservation, functional impact, and biochemical properties [8,9,10,11,12,55,56,57,58]

### 4.3. Performance Evaluation of Predictive Tools

The predictive performance of each tool was evaluated against a ClinVar-based benchmark, in which variants annotated as pathogenic, likely pathogenic, benign, or likely benign served as ground-truth labels and were matched to somatic variants identified in 23 bile duct patients. Performance metrics included area under the curve (AUC), accuracy, precision, recall, and F1-score, which provide insights into the reliability of each prediction tool via the scikit-learn python package [59]. The AUC values, ranging from 0.64 to 1.00, indicate varying levels of effectiveness among the tools. Pathogenicity scoring was performed using a panel of 11 in silico prediction tools (Table 1) For downstream analysis, five tools EVE, REVEL, DEOGEN2, SIFT, and AlphaMissense were selected based on their superior performance across the evaluated metrics. These tools were also chosen for their integration of key biological features, including evolutionary conservation, variation rarity, and protein structural context, which are critical for accurate variant effect prediction. Variants were prioritized if they exceeded a dbNSFP-normalized rankscore ≥ 0.5 in each of the five tools, commonly used pathogenic cut-point for predictors [60].

To explore the potential biological relevance of genes harboring prioritized variants, functional enrichment analysis was performed using g:Profiler [61]. Genes associated with high-confidence variants were subjected to over-representation analysis across multiple curated databases, including KEGG, Reactome, WikiPathways, and the Human Phenotype Ontology [62,63,64,65]. Enrichment significance was determined using a Q-value threshold of 0.05 to control for multiple testing. Statistically significant terms were subsequently visualized using Cytoscape v3.9.1, enabling an interpretable network-based representation of enriched pathways and phenotypic categories [66].

### 4.4. Transcriptomic and Pathway Analysis of Differentially Expressed Genes in Bile Duct Tumors

RNA sequencing (RNA-seq) data from the GSE63420 dataset, comprising seven non-tumor tissues adjacent to tumors and seven bile duct tumor samples, were analyzed together with TCGA dataset, containing total of 28 bile duct tumor sample, and batch effects were corrected using ComBat v3.58.0. ComBat was applied to raw counts from 42 samples (35 cases and 7 controls), modeling study-of-origin as the batch factor (TCGA, *n* = 28, GSE63420, *n* = 14) and preserving the biological signal by including condition (Case vs Control) as the grouping term; library-size normalization (TMM) was used for pre/post correction quality assessment via PCA [67]. Before differential expression, lowly expressed genes were removed using edgeR’s expression-based prefiltering [68]: genes were kept if their CPM exceeded a data-driven threshold, set by a specified minimum read count together with the observed library sizes, in at least n samples, where n is determined by the experimental design (effectively the smallest group size, or more generally the minimum inverse leverage across fitted values). When all groups are sufficiently large, this requirement is slightly relaxed, but n is always maintained above 70% of the smallest group size; for DESeq2, only genes with total counts ≥ 10 across all 42 samples were analyzed. Differentially expressed genes (DEGs) were identified using the DESeq2 v1.46.0 R package [69,70]. Genes with an adjusted *p*-value below 0.05 were considered statistically significant. To explore the functional relevance of these DEGs, gene set enrichment analysis (GSEA) was performed using the clusterProfiler v4.14.6 R package, focusing on KEGG pathway enrichment [71]. Among the significantly enriched pathways, 24 variant-associated genes were further investigated to assess their involvement in key biological processes related to bile duct tumorigenesis.

RNA-seq data were analyzed in R v4.4.3. Using clusterProfiler, KEGG over-representation was visualized with a dotplot that displays pathway significance (−log_10_ adjusted P) and gene counts. Moreover, the overlap of the 24 variant-associated genes was mapped with an UpSet plot. Differential expression was displayed in a ggplot2 v3.5.2 volcano plot, highlighting genes with |log_2_FC| ≥ 1 and FDR < 0.05 [72].

### 4.5. Single-Cell RNA-Seq Analysis of iCCA

Single-cell RNA sequencing (scRNA-seq) data from the GSE138709 [73] were utilized, which includes iCCA tumor and adjacent tissue samples. The data were preprocessed using the scanpy v1.11.3 python package [74].

Cells with a low gene count, fewer than 200 genes detected, were removed to eliminate potential low-quality or dying cells. Additionally, genes expressed in fewer than three cells across the dataset were excluded to reduce noise. To focus the analysis on biologically informative features, highly variable genes (HVGs) were identified based on their expression variability across cells. The top 2000 most variable genes were selected for downstream analysis.

A subset of the data containing only the HVGs was used for dimensionality reduction. Gene expression values were scaled to ensure comparability, with extreme values capped to limit the influence of outliers. Principal Component Analysis was performed to reduce the dimensionality of the data while preserving major sources of variance [75].

Using the principal components, a nearest-neighbor graph was constructed to capture the local structure of the data. This graph was used to compute a two-dimensional embedding using Uniform Manifold Approximation and Projection (UMAP) for visualization of cellular relationships [76]. Clustering was performed using the Leiden algorithm with moderate resolution to identify transcriptionally distinct cell populations [77]. The results of the dimensionality reduction and clustering were integrated back into the full dataset. UMAP plots were generated via scanpy to visualize the distribution of cells by cluster assignment, facilitating interpretation of cellular heterogeneity within the tumor and adjacent tissue microenvironments. To identify cluster-specific marker genes, differential expression analysis was performed based on the Leiden clustering results. Each cluster was compared against all others using the Wilcoxon rank-sum test [74]. This approach enabled the detection of genes that were significantly enriched in specific cell populations, providing insights into the molecular signatures that define each transcriptionally distinct cluster.

### 4.6. Differential DNA Methylation Analysis

A genome-wide DNA methylation analysis was performed using publicly available data (GSE201241) obtained from the Gene Expression Omnibus [78]. The study included 52 iCCA samples and 12 healthy tissue samples profiled using the Illumina Infinium HumanMethylation450K BeadChip array [79]. Raw IDAT files were analyzed. Quality control, preprocessing, and normalization of methylation data were carried out using the minfi v1.52.1 and minfiData v0.52.0 packages [80,81]. Probes with known single nucleotide polymorphisms (SNPs) and those that failed to map reliably to the genome were filtered out to ensure data quality. Statistical modeling to identify differentially methylated positions (DMPs) between iCCA and control groups was performed using the limma v3.62.2 package [82]. Methylation data were converted to M-values for robust statistical inference. Probe annotations, including genomic location, gene association, and regulatory context, were incorporated using the “IlluminaHumanMethylation450kanno.ilmn12.hg19” v0.6.1 annotation package. To identify differentially methylated regions (DMRs), regional analysis was conducted using the DMRcate v3.2.1 and DMRcatedata v2.24.0 packages [83]. This approach aggregates nearby CpG sites showing consistent methylation changes, improving biological interpretability.

Finally, functional enrichment analysis was conducted using the mCSEA v1.26.2 package to evaluate methylation changes across promoter regions [84]. Differential DNA methylation and genomic annotation were supported by the Gviz v1.50.0, RColorBrewer v1.1.3, and stringr v1.5.1 packages [85,86].

### 4.7. Structural Modeling and Molecular Dynamics Simulations

To investigate the structural effects of the p.R49C variation in the MAP2K1, three-dimensional (3D) protein models for both the wild-type and mutant forms were generated. Structural predictions were performed using AlphaFold3, which provided high-confidence atomic-resolution models based on deep learning. The p.R49C variation was introduced into the wild-type structure, and both protein structures were subjected to MD simulations to assess conformational stability and flexibility [87].

Two-dimensional residue–residue interaction maps were generated with LigPlot+ v2.2 for both the wild-type MAP2K1 model and the in-silico p.R49C variant. Each PDB file was cleaned with the accompanying Clean utility and opened in LigPlot+ under its “protein–protein (DIMPLOT)” mode. Interactions were computed by the bundled HBPLUS v3.06 engine using its default Baker-and-Hubbard hydrogen-bond geometry and LigPlot+’s standard window for non-bonded contacts (2.9–3.9 Å between heavy atoms). No manual editing of CONECT records, water molecules, or ligands was performed, ensuring that only intra-chain contacts were plotted [88].

Molecular dynamics simulations were carried out using GROMACS, employing the AMBER99SB-ILDN force field for proteins [89,90]. Protein topologies were generated, and the structures were solvated in a cubic box using the SPC/E water model [91]. Appropriate ions (Na+ and Cl−) were added to neutralize the system. Energy minimization was performed to relax steric clashes and optimize geometry, followed by equilibration under constant volume (NVT) [92]. The production MD simulation was run for 5 nanoseconds with a time step of 2 femtoseconds. Long-range electrostatics were calculated using the Particle Mesh Ewald method, and temperature and pressure were maintained at 300 K and 1 bar [93]. All bonds involving hydrogen atoms were constrained using the LINCS algorithm [94]. After the simulation, structural stability and flexibility were evaluated by calculating the Root Mean Square Deviation (RMSD) and Root Mean Square Fluctuation (RMSF) for both the wild-type and mutant proteins via MDAnalysis v2.9.0 python package [95,96]. These analyses provided insight into conformational dynamics and potential functional consequences of the p.R49C variation in MAP2K1.

Electrostatic surface potentials for the wild-type MAP2K1 model were calculated in PyMOL v3.1.6.1 with its built-in vacuum electrostatics routine, which assigns the programme’s default AMBER-derived partial charges and van-der-Waals radii, solves a smoothed Coulombic interaction field in vacuo on a near-surface grid, and projects the resulting scalar potential onto the solvent-excluded molecular surface. The potentials were symmetrically rescaled to ±59 kT e^−1^ and rendered on a red–white–blue ramp (negative, neutral, positive), with a colour-bar legend inserted automatically. Because the calculation omits explicit solvent and ionic screening, it yields a rapid, qualitative map that highlights local charge clusters; the circled region in the figure demarcates the Arg-49 patch targeted for mutation analysis [97].

Computational mutagenesis was carried out with FoldX v5.0 to quantify the thermodynamic effect of substituting Arg-49 with Cys (R49C). The AlphaFold3 structure was first optimized using the RepairPDB routine under default settings to relieve steric clashes and correct side-chain geometries. The variation was then introduced with BuildModel. 20 replicate runs to ensure convergence. For every build, FoldX recalculated the total free energy using its empirical potential that integrates van der Waals, solvation, electrostatic, and hydrogen-bond contributions. The resulting average ΔΔG kcal mol^−1^ was interpreted [98].

After discarding the first 0.5 ns of each of five independent 50-ns trajectories, RMSD values were block-averaged over 100 ps windows (block length > 5 $\tau_{corr}$). Block means (*n* = 250 per system) were compared with Welch’s *t*-test; significance threshold α = 0.05. Effect size was reported as Cohen’s d. Distributional differences were cross-checked with a two-sample Kolmogorov–Smirnov test [99].

## 5. Conclusions

In summary, our integrative multi-omics analysis of CCA unifies somatic variant annotation, bulk and single-cell transcriptomics, DNA-methylation profiling, and molecular-dynamics modeling to construct a coherent functional narrative in which recurrent pathogenic alterations in *MAP2K1*, *FLNC*, *TUBB3*, and *ABCA1* converge on complementary hallmarks of malignancy. *MAP2K1* mutation-driven MAPK hyperactivation, *FLNC*-mediated cytoskeletal plasticity-enriched desmoplastic remodeling, TUBB3-associated microtubule reprogramming, and *ABCA1*-dependent cholesterol dysregulation collectively foster proliferative signaling, invasive motility, stromal crosstalk, and metabolic adaptation. Given that bile duct cancers are rare malignancies, our study emphasizes the investigation of rare variants based on predicted pathogenicity rather than strict recurrence, incorporating computational pathogenicity prediction tools and benchmarking against ClinVar annotations to distinguish benign from pathogenic alterations. Our findings underscore coordinated transcriptional and epigenetic deregulation. This is evidenced by the concordant down-regulation of *ABCA1* coupled with promoter/shore hypermethylation, context-dependent gene-body hypermethylation with upregulation in *FLNC*, and focal hypomethylation at *MAP2K1* despite its reduced expression. Furthermore, molecular-dynamics evidence provides a mechanistic link for pathway activation, showing that the destabilized mutant MEK1 (*MAP2K1*:p.R49C) connects this structural perturbation to pathway activation. Our analysis identified novel bile duct-associated variations that expand the repertoire of known pathogenic alterations in cholangiocarcinoma and provide new molecular targets for therapeutic intervention. Taken together, these convergent layers of evidence nominate the four genes as plausible driver candidates whose intertwined oncogenic circuitry may illuminate facets of CCA pathogenesis and—if validated—could inform a multidimensional framework for biomarker development and rational combinatorial strategies targeting signaling, stromal, and metabolic vulnerabilities.

### 5.1. Limitation

The epidemiologic rarity of cholangiocarcinoma constrained cohort size, balance, and clinical granularity, limiting power for modest effects and for robust, etiology- or subtype-specific analyses. Absolute case counts remain low in most single-center series, and poor survival further depresses point prevalence; globally, liver and intrahepatic CCA combined show an age-standardized incidence of 8.6 per 100,000 in 2022. Beyond rarity, specimen and data availability posed additional constraints: high-quality treatment-naïve, fresh-frozen tumors with matched non-tumor bile duct tissue, longitudinal sampling, and uniformly processed multi-omic profiles (bulk RNA-seq, scRNA-seq, methylation) with complete clinical covariates (therapy, outcomes) are limited in public repositories [53,73,78].

### 5.2. Future Aspect

Future work should emphasize prospective, multi-institutional validation and mechanistic dissection of the nominated alterations. Orthogonal confirmation could include targeted resequencing in expanded, clinically harmonized CCA cohorts; protein-level assessments (e.g., *TUBB3*/*FLNC*) and locus-specific methylation assays; and isogenic perturbation of sentinel variants (e.g., *MAP2K1* p.R49C) in patient-derived organoids and xenografts using CRISPR base editing to quantify pathway output (p-ERK), microtubule dynamics, cytoskeletal mechanics, and cholesterol efflux (*ABCA1*) with pharmacologic challenge (MEK/ERK inhibitors, taxanes, LXR agonists/statins). Embedding these experiments within biomarker-anchored, hypothesis-driven clinical studies—ideally with spatial/single-cell readouts—will be essential to determine predictive utility and translate multi-omic signals into actionable biomarkers.

## Figures and Tables

**Table 1:** Performance metrics (AUC, Accuracy, Precision, Recall, and F1-score) of various variant pathogenicity predictive tools evaluated against ClinVar annotations.

Model	AUC	Accuracy	Precision	Recall	F1
M-CAP	0.914286	0.421053	1.000000	0.214286	0.352941
MetaRNN	0.871429	0.894737	1.000000	0.857143	0.923077
VEST4	0.866667	0.869565	1.000000	0.833333	0.909091
CADD	0.644444	0.782609	0.842105	0.888889	0.864865
REVEL	0.950000	0.684211	1.000000	0.571429	0.727273
AlphaMissense	0.857143	0.842105	1.000000	0.785714	0.880000
SIFT	0.928571	0.894737	1.000000	0.857143	0.923077
Polyphen	0.857143	0.789474	1.000000	0.714286	0.833333
EVE	1.000000	0.846154	1.000000	0.818182	0.900000
DEOGEN2	0.907692	0.888889	1.000000	0.846154	0.916667
MetaLR	0.892857	0.684211	0.900000	0.642857	0.750000

## Data Availability

All datasets analysed in this study are publicly accessible. Somatic-mutation calls and RNA-seq read counts for cholangiocarcinoma were downloaded from The Cancer Genome Atlas (TCGA) via the GDC portal. Bulk RNA-seq read counts and metadata were obtained from the Gene Expression Omnibus (GEO) under accession GSE63420; single-cell RNA-seq expression matrices were retrieved from GEO accession GSE138709; and DNA-methylation raw data (Illumina HumanMethylation450) were obtained from GEO accession GSE201241.

## Supplementary Materials

The following supporting information can be downloaded at: https://www.mdpi.com/article/10.3390/ijms262311244/s1.

## Abbreviations

The following abbreviations are used in this manuscript:

CCA	Cholangiocarcinoma
iCCA	Intrahepatic cholangiocarcinoma
TCGA	The Cancer Genome Atlas
VUS	Variants of uncertain significance
DEGs	Differentially expressed genes
DMPs	Differentially methylated positions
DMRs	Differentially methylated regions
CAF	Cancer-associated fibroblasts
HDL	High-density lipoprotein
AUC	Area under the curve
UMAP	Uniform Manifold Approximation and Projection
PCA	Principal Component Analysis
GSEA	Gene set enrichment analysis
scRNA-seq	Single-cell RNA sequencing
RNA-seq	RNA sequencing
HVGs	Highly variable genes
RMSD	Root Mean Square Deviation
RMSF	Root Mean Square Fluctuation
MD	Molecular dynamics
FDR	False discovery rate
MAF	Minor allele frequency
SNPs	Single nucleotide polymorphisms
NES	Normalized enrichment score
MAPK	Mitogen-activated protein kinase
GDC	Genomic Data Commons
GEO	Gene Expression Omnibus
KEGG	Kyoto Encyclopedia of Genes and Genomes

## References

[1] Brindley P.J., Bachini M., Ilyas S.I., Khan S.A., Loukas A., Sirica A.E., Teh B.T., Wongkham S., Gores G.J. (2021). Cholangiocarcinoma. Nat. Rev. Dis. Prim..

[2] Chan-On W., Nairismägi M.L., Ong C.K., Lim W.K., Dima S., Pairojkul C., Lim K.H., McPherson J.R., Cutcutache I., Heng H.L. (2013). Exome sequencing identifies distinct mutational patterns in liver fluke–related and non-infection-related bile duct cancers. Nat. Genet..

[3] Statistics About Bile Duct Cancer|Cholangiocarcinoma Stats. https://www.cancer.org/cancer/types/bile-duct-cancer/about/key-statistics.html.

[4] Bray F., Laversanne M., Sung H., Ferlay J., Siegel R.L., Soerjomataram I., Jemal A. (2024). Global cancer statistics 2022: GLOBOCAN estimates of incidence and mortality worldwide for 36 cancers in 185 countries. CA Cancer J. Clin..

[5] Everhart J.E., Ruhl C.E. (2009). Burden of digestive diseases in the United States Part III: Liver, biliary tract, and pancreas. Gastroenterology.

[6] Carotenuto M., Sacco A., Forgione L., Normanno N. (2022). Genomic alterations in cholangiocarcinoma: Clinical significance and relevance to therapy. Explor. Target. Antitumor Ther..

[7] Chen S., Francioli L.C., Goodrich J.K., Collins R.L., Kanai M., Wang Q., Karczewski K.J., Farjoun Y., Banks E., Donnelly S. (2024). A Genomic Mutational Constraint Map Using Variation in 76,156 Human Genomes. Nature.

[8] Frazer J., Notin P., Dias M., Gomez A., Min J.K., Brock K., Gal Y., Marks D.S. (2021). Disease variant prediction with deep generative models of evolutionary data. Nature.

[9] Vaser R., Adusumalli S., Leng S.N., Sikic M., Ng P.C. (2016). SIFT missense predictions for genomes. Nat. Protoc..

[10] Raimondi D., Tanyalcin I., Ferté J., Gazzo A., Orlando G., Lenaerts T., Rooman M., Vranken W. (2017). DEOGEN2: Prediction and interactive visualization of single amino acid variant deleteriousness in human proteins. Nucleic Acids Res..

[11] Ioannidis N.M., Rothstein J.H., Pejaver V., Middha S., McDonnell S.K., Baheti S., Musolf A., Li Q., Holzinger E., Karyadi D. (2016). REVEL: An ensemble method for predicting the pathogenicity of rare missense variants. Am. J. Hum. Genet..

[12] Tordai H., Torres O., Csepi M., Padányi R., Lukács G.L., Hegedűs T. (2024). Analysis of AlphaMissense data in different protein groups and structural context. Sci. Data.

[13] Aubrey B.J., Kelly G.L., Janic A., Herold M.J., Strasser A. (2018). How does p53 induce apoptosis and how does this relate to p53-mediated tumour suppression?. Cell Death Differ..

[14] Zhao M., Mishra L., Deng C.-X. (2018). The role of TGF-*β*/SMAD4 signaling in cancer. Int. J. Biol. Sci..

[15] Parsons D.W., Jones S., Zhang X., Lin J.C.H., Leary R.J., Angenendt P., Mankoo P., Carter H., Siu I.M., Gallia G.L. (2008). An integrated genomic analysis of human glioblastoma multiforme. Science.

[16] Johnson R.L., Rothman A.L., Xie J., Goodrich L.V., Bare J.W., Bonifas J.M., Quinn A.G., Myers R.M., Cox D.R., Epstein E.H. (1996). Human homolog of patched, a candidate gene for the basal cell nevus syndrome. Science.

[17] Nozawa H., Oda E., Ueda S., Tamura G., Maesawa C., Muto T., Taniguchi T., Tanaka N. (1998). Functionally inactivating point mutation in the tumor-suppressor IRF-1 gene identified in human gastric cancer. Int. J. Cancer.

[18] Pylayeva-Gupta Y., Grabocka E., Bar-Sagi D. (2011). RAS oncogenes: Weaving a tumorigenic web. Nat. Rev. Cancer.

[19] Sasaki H., Hikosaka Y., Kawano O., Moriyama S., Yano M., Fujii Y. (2010). MEK1 and AKT2 mutations in Japanese lung cancer. J. Thorac. Oncol..

[20] Hancox R.J., Poulton R., Welch D., Olova N., McLachlan C.R., Greene J.M., Sears M.R., Caspi A., Moffitt T.E., Robertson S.P. (2009). Accelerated, decline, in, lung, function, in, cigarette, smokers, is, assoc, iated with *TP53*/*MDM2* polymorphisms. Hum. Genet..

[21] Turcan S., Rohle D., Goenka A., Walsh L.A., Fang F., Yilmaz E., Campos C., Fabius A.W., Lu C., Ward P.S. (2012). IDH1 mutation is sufficient to establish the glioma hypermethylator phenotype. Nature.

[22] Ni P., Li L., Zhu Y., Du K., Nov P., Wang D., Wang C., Kou Q., Li Y., Zhang Y. (2025). Unveiling the multifaceted role of the FLNC gene: Implications for cancer diagnosis and prognosis. Eur. J. Med. Res..

[23] Takahara K., Hoffman G.G., Greenspan D.S. (1995). Complete structural organization of the human alpha 1(V) collagen gene (*COL5A1*): Divergence from the conserved organization of other characterized fibrillar collagen genes. Genomics.

[24] LaFrance B.J., Roostalu J., Henkin G., Greber B.J., Zhang R., Normanno D., McCollum C.O., Surrey T., Nogales E. (2022). Structural transitions in the GTP cap visualized by cryo-electron microscopy of catalytically inactive microtubules. Proc. Natl. Acad. Sci. USA.

[25] Dooley S.A., Engevik K.A., Digrazia J., Stubler R., Kaji I., Krystofiak E., Engevik A.C. (2022). Myosin 5b is required for proper localization of the intermicrovillar adhesion complex in the intestinal brush border. Am. J. Physiol.–Gastrointest. Liver Physiol..

[26] Mak K.M., Png C.Y., Lee D.J. (2016). Type V collagen in health, disease, and fibrosis. Anat. Rec..

[27] Woodard D.R., Daniel S., Nakahara E., Abbas A., DiCesare S.M., Collier G.E., Hulleman J.D. (2022). A loss-of-function cysteine mutant in fibulin-3 (EFEMP1) forms aberrant extracellular disulfide-linked homodimers and alters extracellular matrix composition. Hum. Mutat..

[28] Eason D.D., Shepherd A.T., Blanck G. (1999). Interferon regulatory factor 1 tryptophan 11 to arginine point mutation abolishes DNA binding. Biochim. Biophys. Acta.

[29] Liu F., Pouponnot C., Massague J. (1997). Dual role of the Smad4/DPC4 tumor suppressor in TGF*β*-inducible transcriptional complexes. Genes Dev..

[30] Pidasheva S., Grant M., Canaff L., Ercan O., Kumar U., Hendy G.N. (2006). Calcium-sensing receptor dimerizes in the endoplasmic reticulum: Biochemical and biophysical characterization of *CASR* mutants retained intracellularly. Hum. Mol. Genet..

[31] Ovchinnikov Y.A., Monastyrskaya G.S., Broude N.E., Ushkaryov Y.A., Melkov A.M., Smirnov Y.V., Malyshev I.V., Allikmets R.L., Kostina M.B., Dulubova I.E. (1988). Family of human Na^+^,K^+^-ATPase genes. Structure of the gene for the catalytic subunit (alpha III-form) and its relationship with structural features of the protein. FEBS Lett..

[32] Voskoboinik I., Strausak D., Greenough M., Brooks H., Petris M., Smith S., Mercer J.F., Camakaris J. (1999). Functional analysis of the N-terminal CXXC metal-binding motifs in the human Menkes copper-transporting P-type ATPase expressed in cultured mammalian cells. J. Biol. Chem..

[33] Pan S., Li T.-J. (2009). PTCH1 mutations in odontogenic keratocysts: Are they related to epithelial cell proliferation?. Oral Oncol..

[34] Gremer L., Merbitz-Zahradnik T., Dvorsky R., Cirstea I.C., Kratz C.P., Zenker M., Wittinghofer A., Ahmadian M.R. (2011). Germline *KRAS* mutations cause aberrant biochemical and physical properties leading to developmental disorders. Hum. Mutat..

[35] Dentici M.L., Sarkozy A., Pantaleoni F., Carta C., Lepri F., Ferese R., Cordeddu V., Martinelli S., Briuglia S., Digilio M.C. (2009). Spectrum of *MEK1* and *MEK2* gene mutations in cardio-facio-cutaneous syndrome and genotype–phenotype correlations. Eur. J. Hum. Genet..

[36] Yuan J., Ng W.H., Tian Z., Yap J., Baccarini M., Chen Z., Hu J. (2018). Activating mutations in MEK1 enhance homodimerization and promote tumorigenesis. Sci. Signal..

[37] Zen Y., Britton D., Mitra V., Pike I., Sarker D., Itoh T., Heaton N., Quaglia A. (2014). Tubulin *β*-III: A novel immunohistochemical marker for intrahepatic peripheral cholangiocarcinoma. Histopathology.

[38] Shroff R.T., King G., Colby S., Scott A.J., Borad M., Goff L., Matin K., Mahipal A., Kalyan A., Javle M.M. (2025). SWOG S1815: A Phase III Randomized Trial of Gemcitabine, Cisplatin, and Nab-Paclitaxel Versus Gemcitabine and Cisplatin in Newly Diagnosed, Advanced Biliary Tract Cancers. J. Clin. Oncol..

[39] Choi J.H., Thung S.N. (2024). Recent advances in pathology of intrahepatic cholangiocarcinoma. Cancers.

[40] Sirica A.E., Gores G.J. (2014). Desmoplastic stroma and cholangiocarcinoma: Clinical implications and therapeutic targeting. Hepatology.

[41] Zhu G., Wang Y., Wang Y., Huang H., Li B., Chen P., Chen C., Zhang H., Li Y., Liu H. (2024). Myofibroblasts derived type V collagen promoting tissue mechanical stress and facilitating metastasis and therapy resistance of lung adenocarcinoma cells. Cell Death Dis..

[42] Subrungruang I., Thawornkuno C., Chawalitchewinkoon-Petmitr P., Pairojkul C., Wongkham S., Petmitr S. (2013). Gene expression profiling of intrahepatic cholangiocarcinoma. Asian Pac. J. Cancer Prev..

[43] Vitali E., Franceschini B., Milana F., Soldani C., Polidoro M.A., Carriero R., Kunderfranco P., Trivellin G., Costa G., Milardi G. (2024). Filamin A is involved in human intrahepatic cholangiocarcinoma aggressiveness and progression. Liver Int..

[44] Qiao J., Cui S.J., Xu L.L., Chen S.J., Yao J., Jiang Y.H., Peng G., Fang C.Y., Yang P.Y., Liu F. (2014). Filamin C, a dysregulated protein in cancer revealed by label-free quantitative proteomic analyses of human gastric cancer cells. Oncotarget.

[45] Qi Y., Xu F., Chen L., Li Y., Xu Z., Zhang Y., Wei W., Su N., Zhang T., Fan F. (2016). Quantitative proteomics reveals FLNC as a potential progression marker for the development of hepatocellular carcinoma. Oncotarget.

[46] Seeree P., Janvilisri T., Kangsamaksin T., Tohtong R., Kumkate S. (2019). Downregulation of ABCA1 and ABCG1 transporters by simvastatin in cholangiocarcinoma cells. Oncol. Lett..

[47] Zhu A.X., Borger D.R., Kim Y., Cosgrove D., Ejaz A., Alexandrescu S., Groeschl R.T., Deshpande V., Lindberg J.M., Ferrone C. (2014). Genomic profiling of intrahepatic cholangiocarcinoma: Refining prognosis and identifying therapeutic targets. Ann. Surg. Oncol..

[48] Quinn L.M., Haldenby S., Antzcak P., Fowler A., Bullock K., Kenny J., Gilbert T., Andrews T., Diaz-Nieto R., Fenwick S. (2023). Genomic profiling of idiopathic peri-hilar cholangiocarcinoma reveals new targets and mutational pathways. Sci. Rep..

[49] Doherty M.K., Tam V.C., McNamara M.G., Jang R., Hedley D., Chen E., Dhani N., Tang P., Sim H.-W., O’Kane G.M. (2022). Randomised, Phase II study of selumetinib, an oral inhibitor of MEK, in combination with cisplatin and gemcitabine chemotherapy for patients with advanced biliary tract cancer. Br. J. Cancer.

[50] Yarchoan M., Cope L., Ruggieri A.N., Anders R.A., Noonan A.M., Goff L.W., Goyal L., Lacy J., Li D., Patel A.K. (2021). Multicenter randomized phase II trial of atezolizumab with or without cobimetinib in biliary tract cancers. J. Clin. Investig..

[51] Heumann T.R., Yarchoan M., Murray J., Lu J., Li D., Kunk P.R., Azad N.S., Kalyan A., Wang H., Sharon E. (2024). A randomized phase 2 study of combination atezolizumab and varlilumab (CDX-1127) with or without addition of cobimetinib in previously treated unresectable biliary tract cancer (ETCTN 10476). J. Clin. Oncol..

[52] Goyal L., Meric-Bernstam F., Hollebecque A., Valle J.W., Morizane C., Karasic T.B., Bridgewater J.A., Wacheck V., He Y., Liu M. (2023). Futibatinib for FGFR2-rearranged intrahepatic cholangiocarcinoma. N. Engl. J. Med..

[53] Weinstein J.N., Collisson E.A., Mills G.B., Shaw K.R., Ozenberger B.A., Ellrott K., Shmulevich I., Sander C., Stuart J.M. (2013). The cancer genome atlas pan-cancer analysis project. Nat. Genet..

[54] Wang K., Li M., Hakonarson H. (2010). ANNOVAR: Functional annotation of genetic variants from high-throughput sequencing data. Nucleic Acids Res..

[55] Jagadeesh K.A., Wenger A.M., Berger M.J., Guturu H., Stenson P.D., Cooper D.N., Bernstein J.A., Bejerano G. (2016). M-CAP eliminates a majority of variants of uncertain significance in clinical exomes at high sensitivity. Nat. Genet..

[56] Li C., Zhi D., Wang K., Liu X. (2022). MetaRNN: Differentiating rare pathogenic and rare benign missense SNVs and InDels using deep learning. Genome Med..

[57] Carter H., Douville C., Stenson P.D., Cooper D.N., Karchin R. (2013). Identifying Mendelian disease genes with the variant effect scoring tool. BMC Genom..

[58] Schubach M., Maass T., Nazaretyan L., Röner S., Kircher M. (2024). CADD v1. 7: Using protein language models, regulatory CNNs and other nucleotide-level scores to improve genome-wide variant predictions. Nucleic Acids Res..

[59] Kramer O. (2016). Machine Learning for Evolution Strategies.

[60] Liu X., Li C., Mou C., Dong Y., Tu Y. (2020). dbNSFP v4: A comprehensive database of transcript-specific functional predictions and annotations for human nonsynonymous and splice-site SNVs. Genome Med..

[61] Reimand J., Arak T., Adler P., Kolberg L., Reisberg S., Peterson H., Vilo J. (2016). g: Profiler—A web server for functional interpretation of gene lists (2016 update). Nucleic Acids Res..

[62] Kanehisa M. The KEGG database. Proceedings of the ‘In Silico’ Simulation of Biological Processes: Novartis Foundation Symposium 247.

[63] Jassal B., Matthews L., Viteri G., Gong C., Lorente P., Fabregat A., Sidiropoulos K., Cook J., Gillespie M., Haw R. (2020). The reactome pathway knowledgebase. Nucleic Acids Res..

[64] Köhler S., Gargano M., Matentzoglu N., Carmody L.C., Lewis-Smith D., Vasilevsky N.A., Danis D., Balagura G., Baynam G., Brower A.M. (2021). The human phenotype ontology in 2021. Nucleic Acids Res..

[65] Martens M., Ammar A., Riutta A., Waagmeester A., Slenter D.N., Hanspers K., Miller R.A., Digles D., Lopes E.N., Ehrhart F. (2021). WikiPathways: Connecting communities. Nucleic Acids Res..

[66] Shannon P., Markiel A., Ozier O., Baliga N.S., Wang J.T., Ramage D., Amin N., Schwikowski B., Ideker T. (2003). Cytoscape: A software environment for integrated models of biomolecular interaction networks. Genome Res..

[67] Leek J.T., Johnson W.E., Parker H.S., Fertig E.J., Jaffe A.E., Storey J.D., Zhang Y., Torres L.C. (2019). sva: Surrogate variable analysis. R Package Version.

[68] Robinson M.D., McCarthy D.J., Smyth G.K. (2010). edgeR: A Bioconductor package for differential expression analysis of digital gene expression data. Bioinformatics.

[69] Sia D., Losic B., Moeini A., Cabellos L., Hao K., Revill K., Bonal D., Miltiadous O., Zhang Z.Y., Hoshida Y. (2015). Massive parallel sequencing uncovers actionable FGFR2-PPHLN1 fusion and ARAF mutations in intrahepatic cholangiocarcinoma. Nat. Commun..

[70] Love M.I., Huber W., Anders S. (2014). Moderated estimation of fold change and dispersion for RNA-seq data with DESeq2. Genome Biol..

[71] Yu G., Wang L.G., Han Y., He Q.Y. (2012). clusterProfiler: An R package for comparing biological themes among gene clusters. OMICS.

[72] Wickham H. (2011). ggplot2. Wiley Interdiscip. Rev. Comput. Stat..

[73] Yao W., Liu X., He Y., Tian M., Lu S., Wang Q., Zheng Y., Lv Z., Hao C., Xue D. (2022). ScRNA-seq and bulk RNA-seq reveal the characteristics of ferroptosis and establish a risk signature in cholangiocarcinoma. Mol. Ther. Oncolytics.

[74] Wolf F.A., Angerer P., Theis F.J. (2018). SCANPY: Large-scale single-cell gene expression data analysis. Genome Biol..

[75] Mackiewicz A., Ratajczak W. (1993). Principal components analysis (PCA). Comput. Geosci..

[76] McInnes L., Healy J., Melville J. (2018). Umap: Uniform manifold approximation and projection for dimension reduction. arXiv.

[77] Traag V.A., Waltman L., Van Eck N.J. (2019). From Louvain to Leiden: Guaranteeing well-connected communities. Sci. Rep..

[78] Goeppert B., Toth R., Singer S., Albrecht T., Lipka D.B., Lutsik P., Brocks D., Baehr M., Muecke O., Assenov Y. (2019). Integrative analysis defines distinct prognostic subgroups of intrahepatic cholangiocarcinoma. Hepatology.

[79] Wang Z., Wu X., Wang Y. (2018). A framework for analyzing DNA methylation data from Illumina Infinium HumanMethylation450 BeadChip. BMC Bioinform..

[80] Fortin J.P., Triche Jr T.J., Hansen K.D. (2017). Preprocessing, normalization and integration of the Illumina HumanMethylationEPIC array with minfi. Bioinformatics.

[81] Hansen K.D., Aryee M., Timp W. minfiData: Example Data for the Illumina Methylation 450 k Array. R Package Version 0.54.0. https://bioconductor.org/packages/minfiData.

[82] Ritchie M.E., Phipson B., Wu D., Hu Y., Law C.W., Shi W., Smyth G.K. (2015). limma powers differential expression analyses for RNA-sequencing and microarray studies. Nucleic Acids Res..

[83] Peters T.J., Buckley M.J., Chen Y., Smyth G.K., Goodnow C.C., Clark S.J. (2021). Calling differentially methylated regions from whole genome bisulphite sequencing with DMRcate. Nucleic Acids Res..

[84] Martorell-Marugán J., González-Rumayor V., Carmona-Sáez P. (2019). mCSEA: Detecting subtle differentially methylated regions. Bioinformatics.

[85] Hahne F., Ivanek R. (2016). Visualizing genomic data using Gviz and bioconductor. Statistical Genomics: Methods and Protocols.

[86] Wickham H., Wickham M.H. (2019). Package ‘Stringr’. https://github.com/tidyverse/stringr.

[87] Abramson J., Adler J., Dunger J., Evans R., Green T., Pritzel A., Ronneberger O., Willmore L., Ballard A.J., Bambrick J. (2024). Accurate structure prediction of biomolecular interactions with AlphaFold 3. Nature.

[88] Wallace A.C., Laskowski R.A., Thornton J.M. (1995). LIGPLOT: A program to generate schematic diagrams of protein-ligand interactions. Protein Eng. Des. Sel..

[89] Abraham M.J., Murtola T., Schulz R., Páll S., Smith J.C., Hess B., Lindahl E. (2015). GROMACS: High performance molecular simulations through multi-level parallelism from laptops to supercomputers. SoftwareX.

[90] Lindorff-Larsen K., Piana S., Palmo K., Maragakis P., Klepeis J.L., Dror R.O., Shaw D.E. (2010). Improved side-chain torsion potentials for the Amber ff99SB protein force field. Proteins.

[91] Campo M.G. (2010). Structural and dynamic properties of SPC/E water. Pap. Phys..

[92] Wall M.E., Calabró G., Bayly C.I., Mobley D.L., Warren G.L. (2019). Biomolecular Solvation Structure Revealed by Molecular Dynamics Simulations. J. Am. Chem. Soc..

[93] Aier I., Varadwaj P.K., Raj U. (2016). Structural insights into conformational stability of both wild-type and mutant EZH2 receptor. Sci. Rep..

[94] Hess B., Bekker H., Berendsen H.J.C., Fraaije J.G.E.M. (1997). LINCS: A linear constraint solver for molecular simulations. J. Comput. Chem..

[95] Carugo O. (2003). How root-mean-square distance (rmsd) values depend on the resolution of protein structures that are compared. J. Appl. Crystallogr..

[96] Michaud-Agrawal N., Denning E.J., Woolf T.B., Beckstein O. (2011). MDAnalysis: A toolkit for the analysis of molecular dynamics simulations. J. Comput. Chem..

[97] Yuan S., Chan H.C.S., Hu Z. (2017). Using PyMOL as a platform for computational drug design. Wiley Interdiscip. Rev. Comput. Mol. Sci..

[98] Buß O., Rudat J., Ochsenreither K. (2018). FoldX as protein engineering tool: Better than random based approaches?. Comput. Struct. Biotechnol. J..

[99] Flyvbjerg H., Petersen H.G. (1989). Error estimates on averages of correlated data. J. Chem. Phys..

